# Irreversible Horner’s syndrome diagnosed by aproclonidine test due to benign thyroid nodule

**DOI:** 10.12669/pjms.291.2732

**Published:** 2013

**Authors:** Coskun M, Aydogan A, Gokce C, Ilhan O, Ozkan OV, Gokce H, Oksuz H

**Affiliations:** 1 Coskun M, Department of Ophthalmology, Mustafa Kemal University, Medical Faculty, Hatay, Turkey.; 2 Aydogan A, Department of General Surgery, Mustafa Kemal University, Medical Faculty, Hatay, Turkey.; 3 Gokce C, Department of Endocrinology, Mustafa Kemal University, Medical Faculty, Hatay, Turkey.; 4 Ilhan O, Department of Ophthalmology, Mustafa Kemal University, Medical Faculty, Hatay, Turkey.; 5 Ozkan OV, Department of General Surgery, Mustafa Kemal University, Medical Faculty, Hatay, Turkey.; 6 Gokce H, Department of Pathology, Mustafa Kemal University, Medical Faculty, Hatay, Turkey.; 7 Oksuz H, Department of Ophthalmology, Mustafa Kemal University, Medical Faculty, Hatay, Turkey.

**Keywords:** Horner’s syndrome, Thyroid nodule, Aproclonidine

## Abstract

We are reporting an irreversible Horner Syndrome (HS) in a patient with benign thyroid gland nodule in which thyroidectomy was performed for treatment. A 37-year-old female was admitted to our clinic with a swelling in the left lobe of the thyroid gland and ptosis at the left eyelid. The clinical diagnosis of HS was confirmed pharmacologically by aproclonidine. Histopathologic examination of thyroidectomy specimen was reported as benign nodule. To the best of our knowledge, this is a very rare report in terms of thyroid benign nodule associated with irreversible HS due to cervical sympathetic chain compression.

## Introduction

 Horner’s syndrome (HS) is a triad of pupillary miosis, eyelid ptosis and anhidrosis. It is due to the disruption of the sympathetic pathways between the eye and the brain.^1^

 Thyroid nodules generally present as an asymptomatic mass at the gland. The differential diagnosis of a thyroid nodule consists of colloid nodule, adenoma, lymphocytic thyroiditis, thyroglossal duct cyst, and malignancies.^2^^-^^6^ Compression of the cervical sympathetic chain by large benign or malignant thyroid nodule rarely may result in HS.^7^^,^^8^

 Here, we present a case diagnosed as irreversible HS due to a benign thyroid nodule. To the best of our knowledge, this is a very rare report regarding thyroid benign nodule associated with irreversible HS due to cervical sympathetic chain compression in the literature. In this report, also, the nodular goiter associated with HS is investigated and the clinical diagnosis of the disease is confirmed via pharmacological testing by aproclonidine.

## Case Report

**fig.1a:**
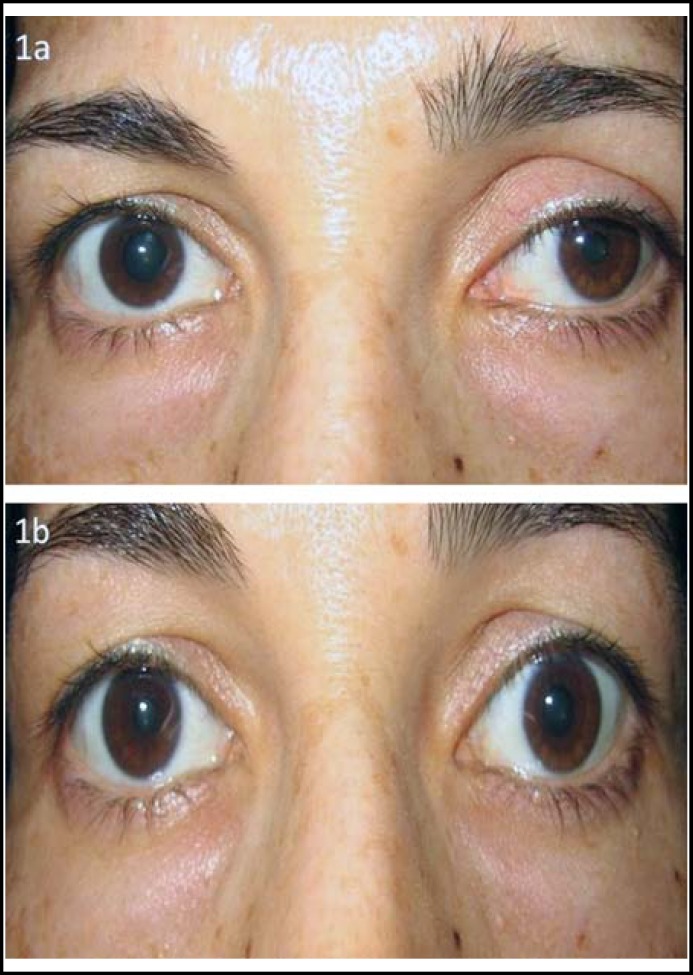
37-year-old female patient with left Horner syndrome. Fig.1b: The resolution of the anisocoria and the left upper lid ptosis after instillation of apraclonidine.

 A 37 years old woman was admitted to the outpatient clinic due to a swelling on her neck, left upper eyelid ptosis and miosis for two years (Fig.1 a, b). There was no palpitation, sweating and nervousness. On physical examination, there was a mass with a diameter of approximately 5 x 5 cm located at the left thyroid lobe and ptosis of the left eyelid. The thyroid functions tests in terms of free T3, free T4, TSH were normal.

**Fig.2a:**
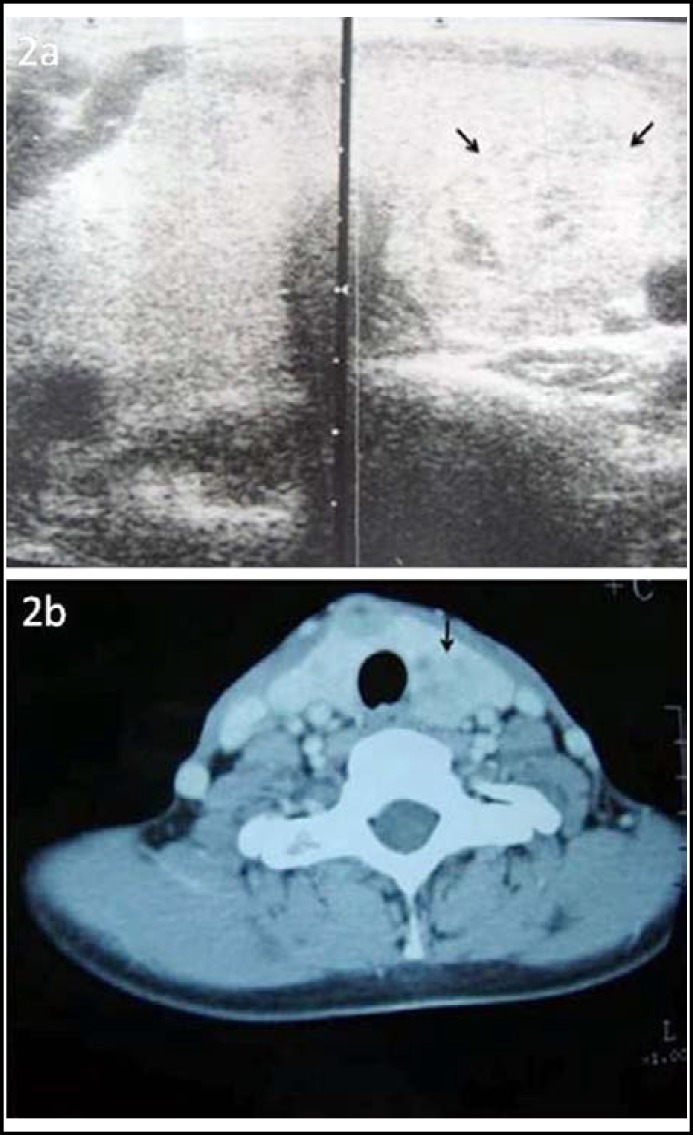
Thyroid ultrasound displaying a solitary heterogeneous nodule which is 55 X 51 mm in size. Fig.2b: Computerize tomography imaging of the neck demonstrating the same thyroid nodule.

 Thyroid ultrasonography displayed that there was a solitary heterogeneous nodule which is 55 x 51 mm in size located at the left upper posterior of the gland. In addition, there were two nodules at the left thyroid lobe. The larger one is hypo-echogenous and has a dimension of 15 x 8 mm. The other nodule has a dimension of 7 x 5 mm, located at the posterior section of the right lobe (Fig.2a). Similar images were also observed in a computerized tomography (Fig.2b). Fine needle aspiration cytology of thyroid nodule was benign in terms of the cytological investigation.

 The patient had consultation the ophthalmology department in aspect of ptosis and miosis. On the ophthalmologic examination (MC), there was left upper eyelid ptosis and miosis. Under the bright conditions, the pupil sizes were measured as 4 mm in the right eye and 2 mm in the left eye. Under the dim light, they were measured as 6 mm in the right eye and 3 mm in the left eye, and there was left pupillary dilatation lag. The pupils were equally responsive to both the light and near stimuli. Extraocular motility was normal in the eyes. No relative afferent pupillary defect was detected. Bilaterally, intraocular pressure was normal. There was no abnormal finding by slit-lamp and at the fundus examinations of both eyes. Best corrected visual acuities were 20/20 in both of the eyes. After instillation of topical aproclonidine (0.5%), (Iopidine, Alcon Laboratories Inc, Fort Worth, Texas) the right pupil was measured as 2 mm, while the left one was measured as 4 mm, the anisocoria reversed and the ptosis was entirely resolved (Fig.1b).

 The patient had thyroidectomy after giving the informed consent. Postoperative surgical specimen was consistent with a benign nodular goiter. There was no improvement in terms of HS during the last 8 months follow- up.

## Discussion

 Horner’s syndrome as a result of thyroid diseases is an uncommon complication. It may be due to benign and malignant thyroid nodules.^7^^,^^8^ It is expected that the syndrome is more likely to develop in patients with malignant thyroid nodules. In the present case, HS had developed even though the thyroidal nodule was benign. In a study, a case with HS due to a benign multinodular goiter was operated and the patient’s symptoms were resolved after the surgery.^7^^-^^9^ In the current report, the case had surgical treatment due to both the large thyroidal nodule and its compression on the sympathetic chains causing HS. However, no improvement in HS was observed in this patient after the surgery, and it is probably related to irreversible changes of the ocular sympathetic nerve. Therefore, it is suggested that the large nodule had been present for a long time, and resulted in irreversible HS for two years due to the nerve compression.

 In previous reports, HS due to thyroidal disorders are not evaluated in detail in case of ophthalmologic investigation such as pharmacological eye tests.^7^^-^^9^ It is necessary to support the diagnosis of HS by pharmacological testing.^10^ If the result of the pharmacological testing is negative, then the diagnosis could be considered as pseudo-HS. Actually, in patients with pseudo-HS, the etiology of symptoms such as ptosis and anisocoria may be due to other diseases rather than HS.^9^ In the current study, the clinical diagnosis was confirmed by pharmacological testing using topical apraclonidine. Topical cocaine and hydroxyamphetamine were also previously used in the diagnosis of HS as pharmacological testing.^10^^,^^11^ Recently, aproclonidine is another drug commonly used to diagnose the HS due to its cost and ease availability.^11^^,^^12^

 Here, we present a case diagnosed as irreversible HS due to a large benign thyroid nodule confirmed by aproclonidine test. To the best of our knowledge, this is a very rare report in aspect of large thyroid benign nodule associated with irreversible HS due to cervical sympathetic chain compression in the literature. Also, after the thyroid surgery, HS was not resolved in comparison to the some previous reports.^7^^,^^8^ In addition, pharmacological testing such as aproclonidine was used for the confirmation of the diagnosis in the current case.

 In conclusion, nodular goiter associated with HS should be investigated and clinical diagnosis should be confirmed via pharmacological testing such as aproclonidine.
